# Reliability of DNA methylation measures using Illumina methylation BeadChip

**DOI:** 10.1080/15592294.2020.1805692

**Published:** 2020-08-15

**Authors:** Zongli Xu, Jack A. Taylor

**Affiliations:** a Epidemiology Branch, National Institute of Environmental Health Sciences, NIH, Research Triangle Park, NC, USA; b Epigenetics & Stem Cell Biology Laboratory, National Institute of Environmental Health Sciences, NIH, Research Triangle Park, NC, USA

**Keywords:** EWAS, methylation array, Illumina, ICC

## Abstract

Illumina BeadChips are widely utilized in epigenome-wide association studies (EWAS). Several studies have reported that many probes on these arrays have poor reliability. Here, we compare different pre-processing methods to improve intra-class correlation coefficients (ICC). We describe the characteristics of ICC across the genome, within and between studies, and across different array platforms. Using technical duplicates from 128 subjects, we find that with raw data only 22.5% of the CpGs on 450 K array have ‘acceptable’ ICCs (>0.5). Data preprocessing steps, such as background correction and dye bias correction, can reduce technical noise and improve the percentage to 38.5%. Similar to previous studies, we found that ICC is associated with CpG methylation level such that 83% of CpGs with intermediate methylation (0.1< beta-value <0.9) have acceptable ICCs, whereas only 21% of CpGs with low or high methylation (beta-value <0.1 or >0.9) have acceptable ICCs. ICC is also correlated with CpG methylation variance; after mutual adjustment for beta-value and variance, only variance remains correlated. Many CpGs with poor ICCs (<0.5) are located in biologically important regulatory regions, including gene promoters and CpG islands. Poor ICC at these sites appears to be a consequence of low biologic variation among individuals rather than increased technical measurement variation. ICCs quality classifications are highly concordant across different array platforms and across different studies. We find that ICC can be reliably estimated with 30 pairs of duplicate samples. CpGs with acceptable ICC have higher study power and are more commonly reported in published epigenome-wide studies.

## Introduction

Illumina DNA methylation BeadChips are widely used in epigenome-wide association studies (EWAS). Array performance is often characterized by examining whole-array correlation from a pair of duplicate samples [1]. Such pairwise whole-array correlations across hundreds of thousands of CpGs are typically very high (e.g., R^2^ > 0.98) [1–3]. However, pairwise whole-array correlations provide virtually no information about measurement reliability at individual CpG sites. For example, if we measured a single CpG twice in 100 individuals but found no correlation between the duplicate measurements, we would be concerned about that site’s reliability. Reliability is a result of both person-to-person variation and technical variation in measurement. Such reliability is often assessed by calculating intra-class correlation coefficients (ICC), a statistic that can use pairs of duplicate samples to quantify ‘biologic variability’ relative to the ‘total variability’ (biologic plus technical variation) [4–6]. Several previous studies showed that majority of the probes on the Illumina methylation BeadChips have low reliability, and the magnitude of reliability is correlated with methylation level and variation [5,6].

Here we use a large set of duplicate samples measured on Illumina arrays to explore the distribution of ICC values, evaluate different data preprocessing methods that can improve ICC, and describe the relationships between ICC, methylation level, variance, and genomic context. We examine the consistency of ICC estimates both within and across different Illumina platforms and across studies. We also determine the number of technical duplicates required to accurately characterize the reliability of a CpG. Finally, we characterize the set of published EWAS CpGs in terms of ICC measurements from our study.

## Result

**Figure 1.**
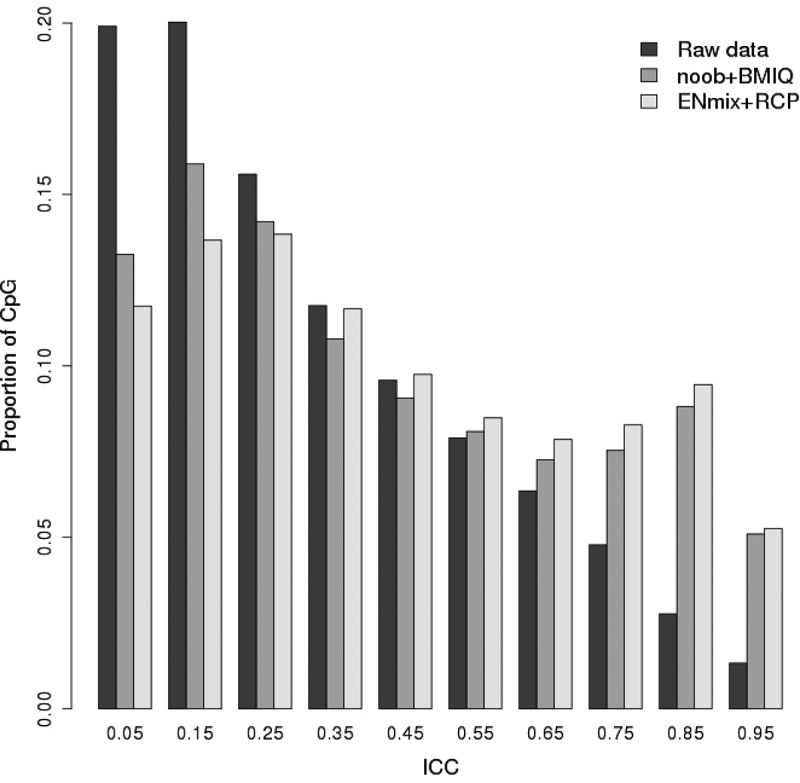
Comparison of ICC values for raw and preprocessed data. Shown are the ICCs calculated based on the 128 technical replicates with 450 K methylation data. Data preprocessing steps include either ENmix background correction, RELIC dye-bias correction, quantile normalization and RCP probe type bias correction (ENmix+RCP) or noob background correction, Illumina dye-bias correction, quantile normalization and BMIQ probe type bias correction (noob+BMIQ). The x-axis labels are the ICC median of each bin

Whole-array Pearson correlation of methylation beta values between each of the Sister Study 128 duplicate pairs was high (Avg R^2^ = 0.99, SD = 0.01, Supplementary Figure 1a). But whole-array correlations are also high when comparing any two unrelated women (Avg R^2^ = 0.98, SD = 0.01, Supplementary Figure 1b), suggesting that simple whole-array correlation is a poor metric for distinguishing duplicates from non-duplicates. If the methylation value of each probe is first centred by its average value among all individuals, then correlation (termed ‘centered correlation’) between duplicates from the same person (Avg centred correlation R^2^ = 0.64, SD = 0.18) are substantially higher than that between two unrelated women where, as expected, the centred correlation approaches zero (Avg centred correlation R^2^ = 0.004, SD = 0.15, Supplementary Figure 1c and 1d). However, neither of these array-wide measures provide information on the reliability of individual CpG probes. To assess individual CpG reliability we first used raw methylation data from 128 duplicates to calculate ICC for each CpGs on the 450 K array and found that only 106,817 (22.5%) had ‘acceptable’ reliability, i.e., with ICC > 0.5 (Figure 1). Preprocessing of raw data with the ENmix pipeline (using default settings) provided substantial improvements to ICC values so that the number of CpGs having ICC > 0.5 increased to 182,234 (38.5%) (Figure 1 and Supplementary Figure 2). Preprocessing by commonly used alternative methods of noob background correction, Illumina dye-bias correction, and BMIQ probe-type bias correction resulted in a smaller percentage (36.7%) of CpGs with acceptable reliability (Figure 1 and Supplementary Figure 2). Examination of both preprocessing routes revealed that the improvements in ICC were due to the background correction and dye-bias correction steps in each route. Although quantile normalization and probe-type bias correction can improve some other data characteristics, they did not improve the overall ICC distribution. For all subsequent analyses, we use ICC values obtained after ENmix preprocessing.

**Figure 2.**
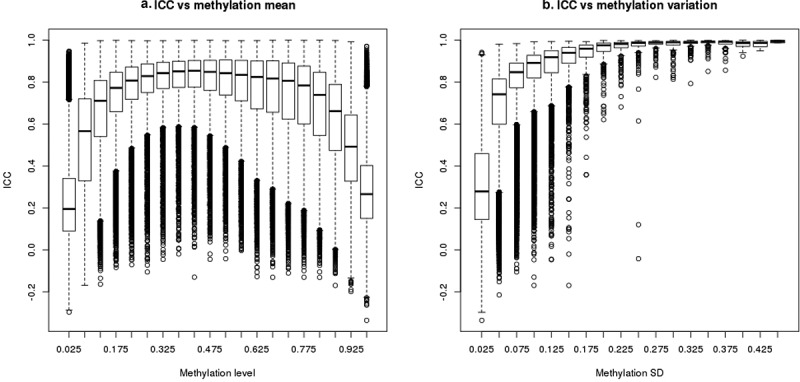
Correlation between ICC and methylation mean or methylation variation. ICC was calculated based on the 128 replicates with 450 K array data for 469,291 CpGs. Methylation standard deviation or mean for each CpG was calculated in 2878 Sister Study samples. Boxplots represent distribution of CpGs by (a) methylation level (beta value) and (b) by methylation SD. The bars represent medians, boxes represent first and third quartiles, whiskers 1.5 inter-quantile range (IQR), and circles are outliers

ICC values are correlated with both average methylation (beta value) and variance. 450 K array blood DNA beta values have a strong bimodal distribution with about 72% of CpGs having either low (<0.1) or high (>0.9) beta values. CpGs with such low or high methylation predominantly have ICC < 0.5 (Figure 2(a)). Only 28% of CpGs on the array have intermediate beta values (between 0.1 and 0.9), and these CpGs have much higher ICC values: 83% having ICC ≥ 0.5 (Figure 2(a)). The majority (87%) of CpGs on the array have low variation (SD < 0.05) in methylation values and most (70%) of these CpGs have poor reliability (Figure 2(b)). In contrast, almost all (95%) of the CpGs with SD ≥ 0.05 have acceptable reliability (Figure 2(b)). Methylation beta value and SD are correlated with one another: most CpGs with low or high beta values (<0.1 or >0.9) have relatively smaller SDs, whereas most CpGs with intermediate beta values have larger SDs (Supplementary Figure 3). After adjusting for SD percentile as a categorical variable, ICC is independent of beta value magnitude; conversely, after adjusting for beta value percentile, ICC remains positively correlated with SD (Supplementary Figure 4). Logit transformation of methylation beta values (termed ‘M values’) is sometimes used to mitigate the correlation between methylation level and methylation variation. However, we find that ICCs calculated using M values are similar to ICCs based on methylation beta values (Supplementary Figure 5), and they remain highly correlated with variance (data not shown). Finally, we examined ICC relative to genomic region. Although CpGs with low ICC were more common in islands (compared to shore, shelf or non-island regions) and within 200bp of transcription start sites (compared to 5ʹUTR, TSS1500, gene body, 3ʹUTR, and other genomic contexts), no differences remained after adjustment for methylation SD (Supplemental Figure 6).

**Figure 3.**
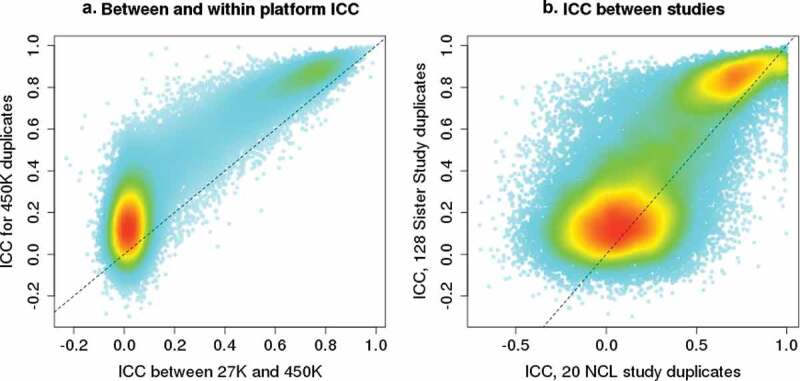
ICC comparisons between array platforms and between different studies. A) Comparison of between- and within-array ICC values for the 25,948 CpGs shared between the Illumina 27 K and 450 K arrays. Within-array ICC plotted on the Y-axis was calculated based on results from 128 duplicates run on 450 K arrays. Between-platform ICC plotted on the X-axis was calculated based on results from 476 Sister Study samples that were assayed on both 27 K and 450 K arrays. B) Comparison of ICC values for CpGs estimated in different studies using 450 K arrays: Sister Study ICC calculated using 128 duplicates from women with an average age of 58 years. The NCL study ICC was calculated using 20 duplicates from newborns, 13 males and 7 females

We examined whether the ICC estimates from 450 K arrays were similar to those calculated across different array platforms and to those from a different study population. Four hundred and seventy-six Sister Study participants were assayed with both 27 K and 450 K arrays, and 25,948 CpGs are represented on both platforms. For these CpGs, the within-450 K array ICCs are correlated with those calculated across-platforms, with stronger correlation for CpGs that have ICC greater than 0.5 (Figure 3(a)). We also examined whether the within-450 K array ICC estimates were replicable across studies by comparing ICC values from the Sister Study (128 duplicates from women with an average age of 58 years), and the NCL study (20 duplicates from 13 male and 7 female newborns). Here again, the majority of CpGs with poor reliability in one study also had poor reliability in the other, whereas the majority of CpGs with ICCs greater than 0.5 in one study showed similarly high ICC values in the other (Figure 3(b)).

**Figure 4.**
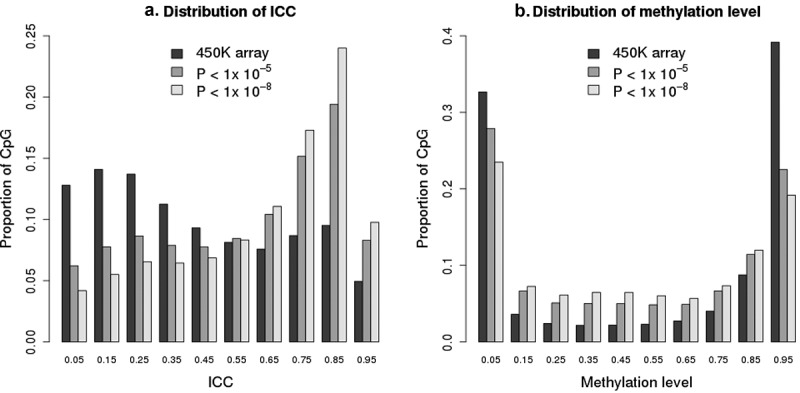
Distribution of all probes on 450 K array compared to CpGs reported as statistically significant in the EWAS Atlas of 570 studies (115,888 CpGs at threshold of p < 10^−5^ or 74,411 CpGs at threshold of p < 10^−^[8]). A) Distributions by ICC values. B) Distributions by methylation (beta) values

We assessed whether CpGs having acceptable reliability in our data were more likely to appear in published EWAS studies. The EWAS Atlas [7] summarizes results from more than 570 studies, and of those CpGs represented on the 450 K array, 115,888 CpGs have reported association P values < 10^−^[5], and 74,411 have reported association P values < 10^−^[8]. Compared to all CpGs on the array where 38.5% had acceptable ICC reliability values, the CpGs reported in the EWAS Atlas are more likely to have had acceptable ICC reliability (of CpGs in the EWAS Atlas reported to have P < 10^−^[5], 62% had acceptable reliability values in our study; of those with P < 10^−^[8], 71% had acceptable reliability) (Figure 4(a)). Compared to all CpGs on the array, CpGs reported in the EWAS Atlas are more likely to have intermediate methylation levels and less likely to have methylation levels less than 0.1 or greater than 0.9 (Figure 4(b)).

In order to calculate ICC, investigators must incur increased costs by measuring duplicate samples. To determine how many duplicates are needed to reliably estimate ICC, we compared ICCs obtained using smaller numbers of duplicate pairs to those from the full set of 128 Sister Study duplicate pairs. Using a threshold ICC = 0.5 to classify CpGs as having poor or acceptable reliability, we found that 30 or more duplicate pairs provided good concordance (>90%) to the reliability classification obtained with the full set (Supplementary Figure 7).

## Discussion

Duplicate samples can provide useful information on the replicability of array-based methylation measurements. Although often used as a first measure of quality control, we found that whole-array correlations between duplicate pairs are very similar to correlations between unrelated sample pairs. This is the consequence of both the bimodally distributed methylation values (close to 0% or to 100%) across CpGs on the array and relatively small variance for any given CpGs across samples. Centred correlation can mitigate the problem and better distinguish duplicate pairs from unrelated sample pairs. However, whole-array correlations provide little insight into population and experimental measurement variability at individual CpG sites.

Using raw data from a large number of duplicate pairs, we found that less than a quarter of the CpGs on 450 K array have ICC values with acceptable reliability, where the biologic variance between individuals is greater than the experimental variance. We found data preprocessing methods, such as background correction and dye bias correction, can reduce experimental variation and substantially increase the number of CpGs with acceptable reliability. After preprocessing, we found that the median ICC for CpGs on the array was 0.41, a value that is similar to that previously reported (0.42) by Dugué et al. [5] for peripheral blood mononuclear cells (PBMC) from Melbourne Collaborative Cohort Study, but higher than the median ICC for duplicates from the Atherosclerosis Risk In Communities (ARIC) Study (0.30) [8].

Population variation is the principal factor determining site-specific ICC values. Although they represent less than a third of the total CpGs on the array, CpGs with intermediate levels of methylation (those with beta values in the range 0.1–0.9) have higher variation among individuals and consequently, almost all have acceptable reliability. In contrast, more than two thirds of CpGs on the 450 K array are either nearly unmethylated or completely methylated in blood DNA. Methylation state at these sites is remarkably consistent across different individuals so that biologic variation is low; consequently, the majority of these sites have poor reliability – a finding similar to that reported by Dugué et al. [5]. Similarly, CpGs located in island regions have lower ICC than shore, shelf, and non-island regions and CpGs located within 200bp of transcription start sites have lower ICC than other genomic locations. Low biologic variation at island and TSS locations is consistent with the hypothesis that methylation is highly constrained in regions important to transcriptional regulation.

ICC reliability classifications of most CpG sites appear to be robust both across platforms and across different populations. Using samples that were assayed with both 27 K and 450 K platforms we found that vast majority of the CpGs classified as having poor reliability based on comparisons of the measurements across the two platforms also had poor reliability based on duplicate sample measurements using the 450 K array. Similarly, we found that the reliability classification obtained from adult women in the U.S.-based Sister Study using the 450 K array were largely concordant to those of male and female infants from Norway using the 450 K array. We interpret these findings as further support for the use of ICC = 0.5 as a threshold for acceptable reliability of CpG methylation values.

Estimation of ICC requires additional costs to assay duplicate samples, an expense that investigators would like to minimize. In line with general recommendations for calculating ICC, our simulations suggest that 30 duplicate samples provide reasonably good agreement with ICC classifications obtained from a much larger sample. To facilitate calculation of ICC, we have added a function ‘dupicc’ to the free ENmix R software package along with centred correlation [9] and other measures that investigators may find useful in assessing and improving the reliability of their data.

CpGs with poor reliability have relatively little power [4,10] to detect weak effects, and we found that they were less likely to be reported in EWASs. Some authors have suggested exclusion of low reliability CpGs from association analyses [4,8]. Given our finding that reliability classifications are robust across studies, *a priori* exclusion of CpGs with known poor reliability has the appealing features of reducing multiple comparisons, decreasing false positives, and increasing power. But we are reluctant to suggest such exclusion for several reasons: Poor reliability is not a consequence of increased experimental or measurement variation at these CpG sites, but rather because of low biologic variation. Excluding such sites would also potentially exclude important gene regulatory regions and risk missing unique ethnic, exposure, or disease effects. Studies will require larger sample sizes, or substantially improved array measurement precision to investigate such biologically important but low-variability sites.

## Method

### Evaluation datasets

We evaluated methylation measurement reliability using duplicate samples from two studies: The Sister Study and the Norway Facial Cleft Study (NCL). The Sister Study is a prospective cohort study of 50,884 women recruited from the United States and Puerto Rico between 2003 and 2009 [11]. Written informed consent and blood samples were collected at recruitment and the study was conducted in accordance with recognized ethical guidelines and approved by the institutional review boards of the National Institute of Environmental Health Sciences (NIEHS), National Institutes of Health (NIH), and the Copernicus Group. We have previously reported the blood DNA methylation in two separate studies, the first study assayed 910 women using the Illumina 27 k array [12], and the second study assayed 2878 women using the Illumina 450 K array [13]. Four hundred and seventy-six women were assayed in both studies. Within the 450 K study, we included technical duplicates from 128 women. The average age at blood draw for women with technical duplicates in both studies was 57.6 (range from 36.6 to 75.1). At the time samples were assayed, technical duplicates were randomly distributed among arrays and the two duplicates of a sample were always measured on different arrays.

We also assayed technical duplicates from 20 participants (13 males and 7 females) in the Norway Facial Cleft Study (NCL) using Illumina 450 K arrays [14]. Blood samples from a total of 898 babies were assayed in this study using heel-stick blood samples that were collected 2–3 days after delivery. Parents gave informed consent for data and sample collection, and all data were anonymized after collection. Ethical approval for this study was obtained from the Norwegian Data Inspectorate, the Regional Research Ethics Committee for Western Norway, and the Institutional Review Board of NIEHS, NIH. In both studies, two samples in a duplicate pair were always assigned to different arrays.

## Methylation analysis

Genomic DNA was extracted from aliquots of whole blood using an automated system (Autopure LS, Gentra Systems). One microgram of DNA from each subject was bisulphite-converted in 96-well plates using the EZ DNA Methylation Kit (Zymo Research, Orange County CA) and methylation analyses for all studies were carried out at the NIH Centre for Inherited Disease Research at Johns Hopkins University (Baltimore, MD). Samples were tested for completion of bisulphite conversion, and converted DNA was analysed on Illumina 27 K Array or HumanMethylation450 Beadchip following the manufacturer’s protocols. The arrays were analysed with high throughput robotics to minimize batch effects.

## Preprocessing

We pre-processed raw methylation data using two different sets of methods: 1) using the ENmix R package software pipeline providing ENmix background correction [9], RELIC dye-bias correction [15], quantile normalization and RCP probe-type adjustment [16], termed as ENmix+RCP or 2) noob background correction [17], Illumina dye-bias correction, quantile normalization, and BMIQ probe-type bias correction [18]. We removed low quality data points (detection P ≥ 10^−6^ or number of beads ≤ 3) and excluded probes with greater than 5% of low-quality data or samples with bisulphite conversion control intensity less than 4000.

## Statistical analysis

The intraclass correlation coefficient (ICC) is commonly used to evaluate reproducibility or reliability of a measurement. ICC values less than 0.5 are usually classified as having poor reliability, whereas those between 0.5 and 0.75, between 0.75 and 0.9, and greater than 0.9 are classified, respectively, as having moderate, good, and excellent reliability [19]. For simplicity, we use the term ‘poor’ reliability for those with ICC ≤ 0.5 and ‘acceptable’ reliability for all those with ICC > 0.5. ICC was calculated with a two-way mixed model using the R package ‘irr’[20] to evaluate measurement consistency ICC(3,1) [21] between duplicate pairs. The equation used in the model [20] can result in ICCs with negative values, which indicate the true ICC is close to 0. ICC can be thought of as the ratio of the biologic variation relative to the total variation (biologic plus experimental measurement variation), with the cut-point of ICC = 0.5 being the point where biologic and experimental variation are approximately equal.

## Supplementary Material

Supplemental Material
