# Supplementary material for: Reliability of DNA methylation measures using Illumina methylation BeadChip
Source: Epigenetics. 2020 Aug 15;16(5):495–502. doi: 10.1080/15592294.2020.1805692 (PMC8078668; doi:10.1080/15592294.2020.1805692)

**Supplementary Figure 1**. Examples of pairwise comparison of raw DNA methylation beta values across all CpG probes on 450k array between: A) replicate pair from same individual; B) samples from two unrelated women; C) replicate pair from same individual using centered methylation values; D) samples from two unrelated women using centered methylation values.

**
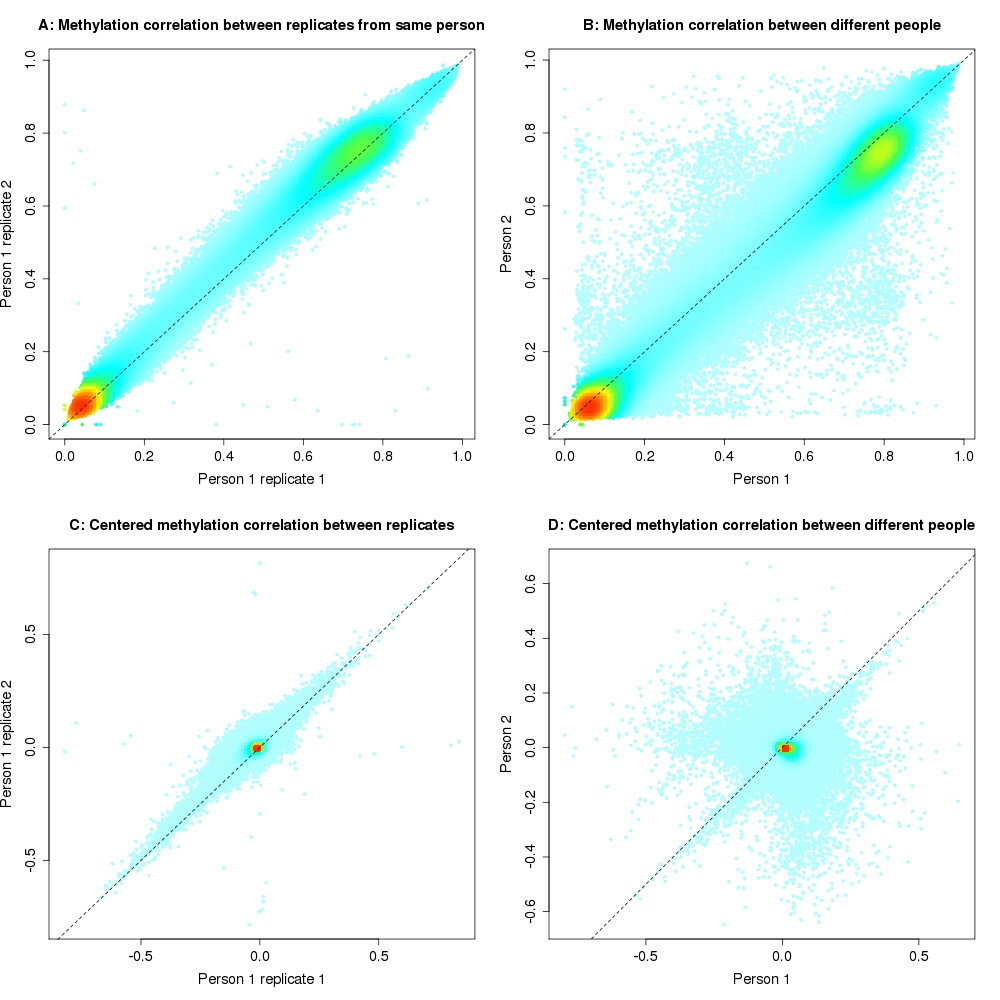
**

**Supplementary Figure 2.** Pairwise comparisons of ICC values obtain using raw data or preprocessed data. Shown are the ICCs calculated based on the 128 technical replicates with 450K methylation data. Data preprocessing were either ENmix background correction, RELIC dye-bias correction and RCP probe type bias correction (ENmix+RCP) or noob background correction, Illumina dye-bias correction and BMIQ probe type bias correction (noob+BMIQ). Using raw data, 22.5% of CpGs on the array had acceptable ICC > 0.5 vs 38.5% using ENmix+RCP preprocessing vs 36.7% using noob+BMIQ.

**
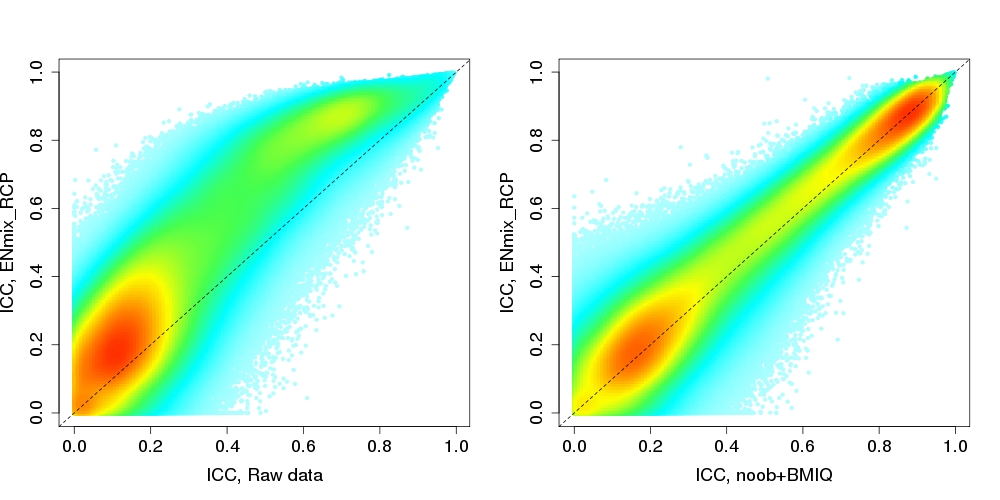
**

**Supplementary Figure 3.** Methylation standard deviation vs methylation level (beta value). 450K data based on 2878 Sister Study samples.


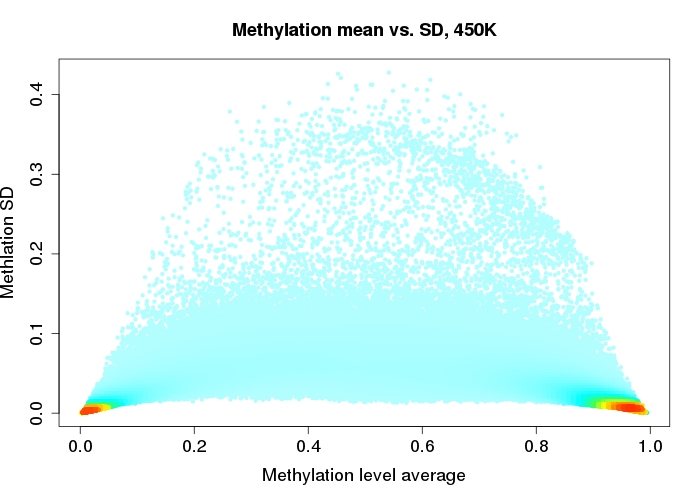


**Supplementary Figure 4.** Methylation level (beta value) or standard deviation (SD) adjusted ICC vs Methylation level/SD. A) methylation level vs ICC; B) Methylation SD vs ICC; C) ICC adjusted for SD vs methylation level; D) ICC adjusted for methylation level vs methylation SD. All ICCs were calcuated based on the 128 replicates and 469291 CpGs. Means (points) and standard deviation (lines) are shown for each of 20 equal-numbered quantiles (vigintiles) of CpGs.


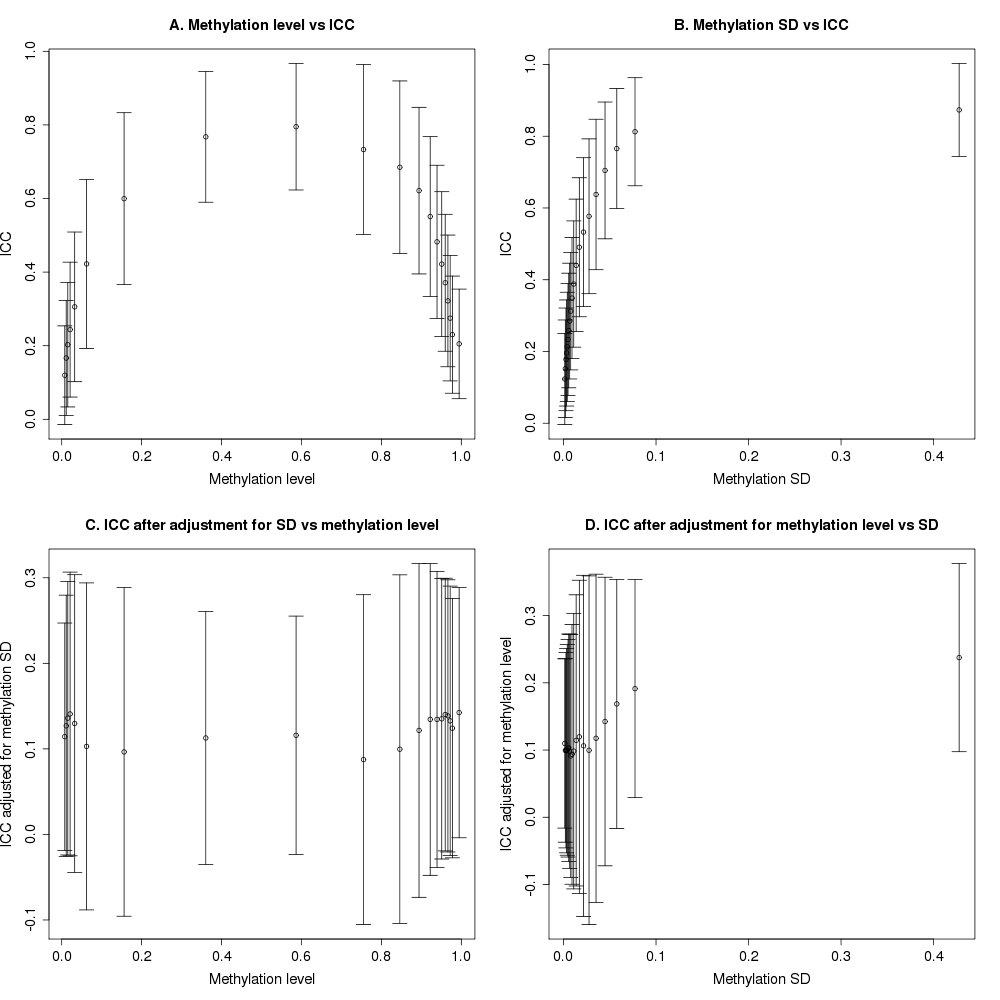


**Supplementary Figure 5.** ICC calculation comparisons between Methylation beta value and logit transformed (M value) methylation beta value. Calculations were done in 128 replicates with 450K data for 469291 CpGs.


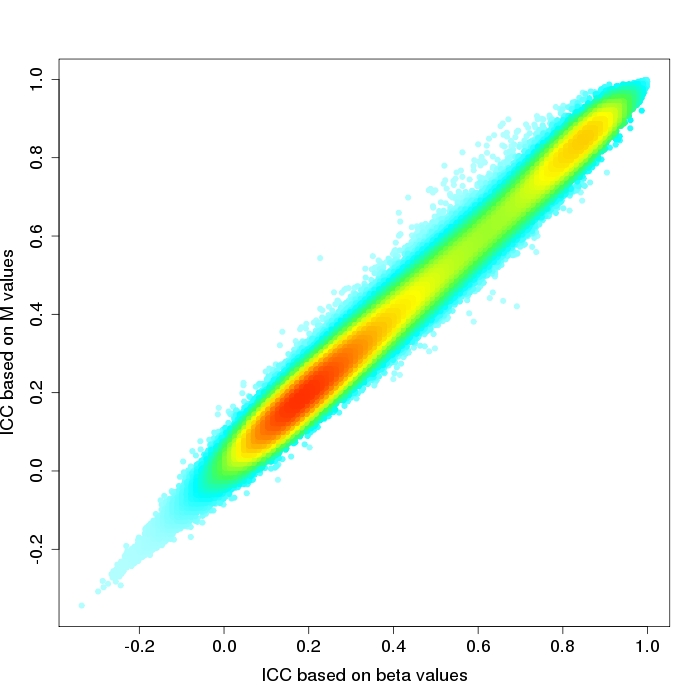


**Supplementary Figure 6.** ICC distributions in different genomic regions. ICC was calculated based on the 128 Sister Study replicates with 450K data for 469291 CpGs. Panel A is shows ICC distributions relative to CpG island locations with panel B showing ICC after adjustment for methylation SD for those same regions. Panel C shows ICC vs genomic locations with panel D showing ICC after adjustment for methylation SD for those same regions.


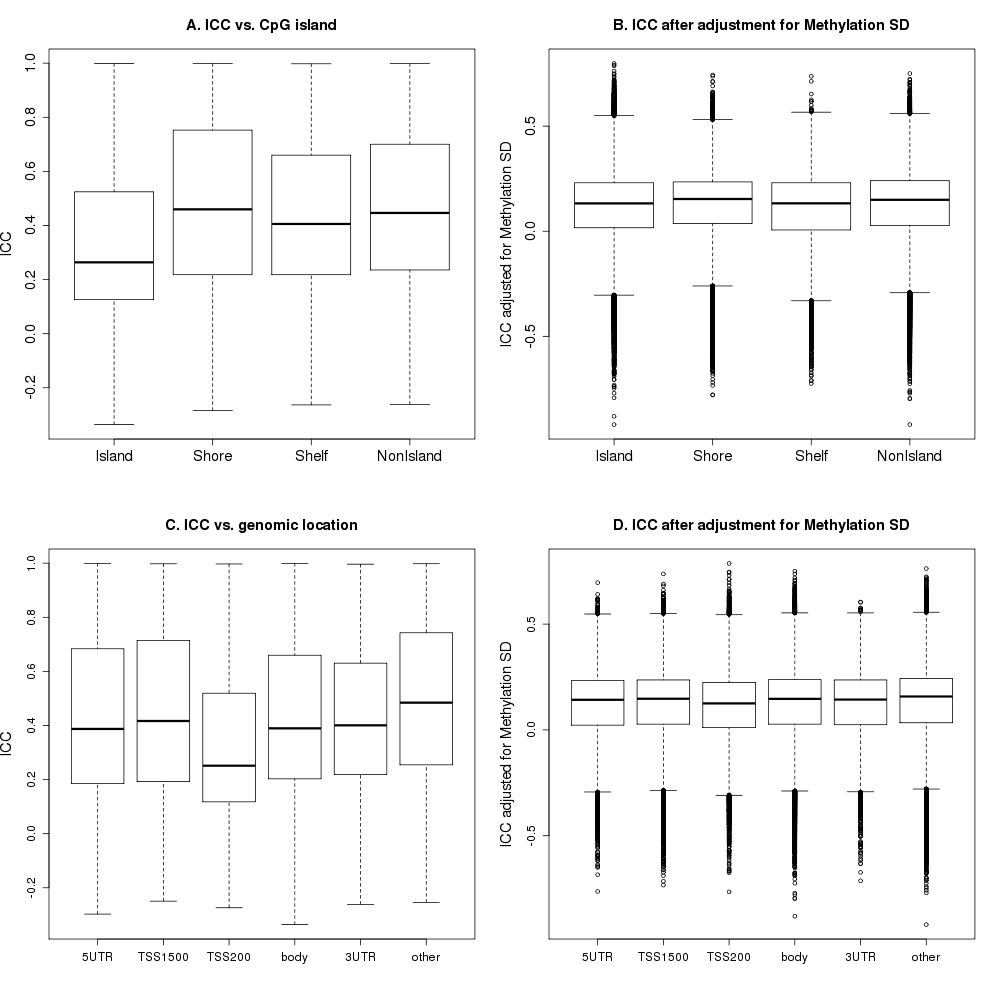


**Supplementary Figure 7**. Concordance of ICC classification (at threshold of 0.5) between full set (128) of replicates and random sample of smaller set of replicates. ICC was calculated in 450K Sister Study data for 469291 CpGs.


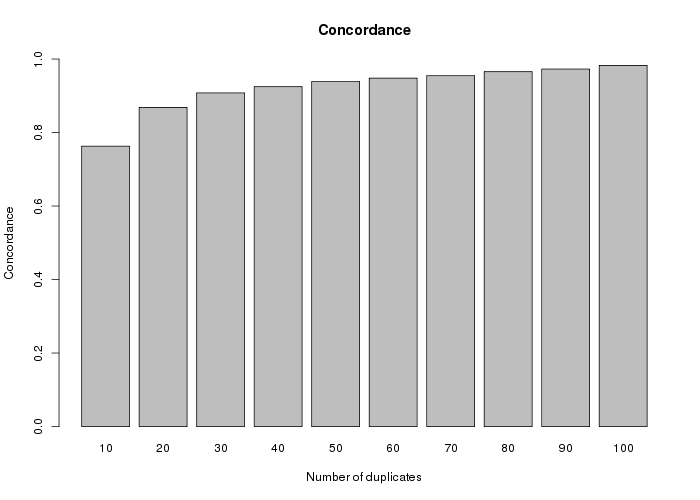

Supplement: Supplemental Material [file KEPI_A_1805692_SM0338.docx]
